# Orthodontic Treatment Planning based on Artificial Neural Networks

**DOI:** 10.1038/s41598-018-38439-w

**Published:** 2019-02-14

**Authors:** Peilin Li, Deyu Kong, Tian Tang, Di Su, Pu Yang, Huixia Wang, Zhihe Zhao, Yang Liu

**Affiliations:** 1 State Key Laboratory of Oral Diseases & National Clinical Research Center for Oral Diseases & Department of Orthodontics, West China Hospital of Stomatology, Sichuan University, Chengdu, 610041 P.R. China; 2 State Key Laboratory of Electronic Thin Films and Integrated Devices, University of Electronic Science and Technology of China, Chengdu, 610054 P. R. China

## Abstract

In this study, multilayer perceptron artificial neural networks are used to predict orthodontic treatment plans, including the determination of extraction-nonextraction, extraction patterns, and anchorage patterns. The neural network can output the feasibilities of several applicable treatment plans, offering orthodontists flexibility in making decisions. The neural network models show an accuracy of 94.0% for extraction-nonextraction prediction, with an area under the curve (AUC) of 0.982, a sensitivity of 94.6%, and a specificity of 93.8%. The accuracies of the extraction patterns and anchorage patterns are 84.2% and 92.8%, respectively. The most important features for prediction of the neural networks are “crowding, upper arch” “ANB” and “curve of Spee”. For handling discrete input features with missing data, the average value method has a better complement performance than the *k*-nearest neighbors (*k-*NN) method; for handling continuous features with missing data, *k-*NN performs better than the other methods most of the time. These results indicate that the proposed method based on artificial neural networks can provide good guidance for orthodontic treatment planning for less-experienced orthodontists.

## Introduction

Malocclusion is a common disease that impairs occlusal function, increases the incidence of caries, causes psychological discomfort, endangers health and reduces the quality of life^1–3^. An epidemiologic survey in America showed that 57% to 59% of each racial group has at least some degree of orthodontic treatment need^4^. The Health Policy Institute of the American Dental Association reported that 33% of young adults avoid smiling due to the condition of their mouth and teeth, and 82% of adults believe that the good appearance of the mouth and teeth can help them advance in life^5^. To achieve satisfactory orthodontic treatment effects, treatment planning must be carefully performed before the treatment process begins^6^. Comprehensive and deliberate evaluation of many factors makes treatment planning a complex process without any objective patterns, and heavily depends on the subjective judgment of the orthodontists.

Researchers have attempted to make orthodontic treatment planning procedures more objective by using some prediction methods. Rule-based expert systems (RBESs) were used to help orthodontic students and inexperienced practitioners with problem-solving and decision-making^7^. RBESs use formulated rules to construct a decision tree but suffer from considerable knowledge lost in the rule determination. To overcome the limitations of RBESs, case-based expert systems (CBESs) have been developed. CBESs acquire new knowledge by analyzing and taking in new cases, thereby acquiring better indexing features^7^. The difficulty of using CBES lies in finding an exact case that matches the new case; thus, some new cases have to be properly modified to be identified. A software that combined RBES and CBES was proposed in Noroozi’s work^8^, and the application of fuzzy logic made it more practical. Takada^9^ and Yagi^10^ proposed a CBES that used a *k*-nearest neighbors (*k-*NN) algorithm to perform classification in tooth-extraction decisions. However, the *k-*NN algorithm is a type of instance-based learning that is sensitive to the local structure of data and requires an increasing number of calculations as the number of cases increases. The artificial neural network (ANN) has the advantage of excavating features from massive medical data^11,12^, and the past decade has witnessed the rapid development of this approach. It has also been applied to determine necessary tooth extraction^13^ and extraction patterns (specific teeth to be removed)^14^ in orthodontic treatment planning.

It is important to note that different orthodontists can have markedly different plans for a specific case^15^. Considerable variety can occur particularly in the decision of which teeth to extract^16^. In addition to outputting a recommended treatment plan, an ANN that can output the feasibilities of multiple extraction options will allow orthodontists greater flexibility. Additionally, anchorage (resistance to unwanted tooth movement) is another important factor that should be considered when making plans^6^. In extraction cases using maximum anchorage, the early use of appropriate means to reinforce anchorage must be taken into account in the beginning. Related data are not always available in the actual application environment of ANNs^17–19^. ANN models cannot predict missing data; and thus a case with incomplete data may not be predicted by the models^20–22^. Providing methods that can handle missing data may make the model more applicable. Statistical approaches, such as imputation with average value or normal value, and *k-*NN imputation method have been intensively studied^22,23^. However, the comparison of different methods have showed different results and there is not a unique solution that can obtain best results in each neural network^18,21,24^. In this study, the traditional statistical approaches (average value method, frequent value method, specific value method and median value method) and the *k-*NN method are used to evaluate which method may be the best for increasing the accuracy.

The ANN in this study can promptly output both a recommended plan and feasible probabilities of several alternative options. The plan covers the most crucial concerns of orthodontists for most cases, including the extraction-nonextraction decision, extraction patterns and anchorage patterns (whether to use maximum anchorage to retract anterior teeth). We further calculated the relative contribution of features in each network model with the partial derivatives (PaD) method^25^ and, for the first time, investigated the effect of several complementary methods on handling missing data in orthodontic treatment planning.

## Results

### Orthodontic treatment planning with ANNs

**Figure 1:**
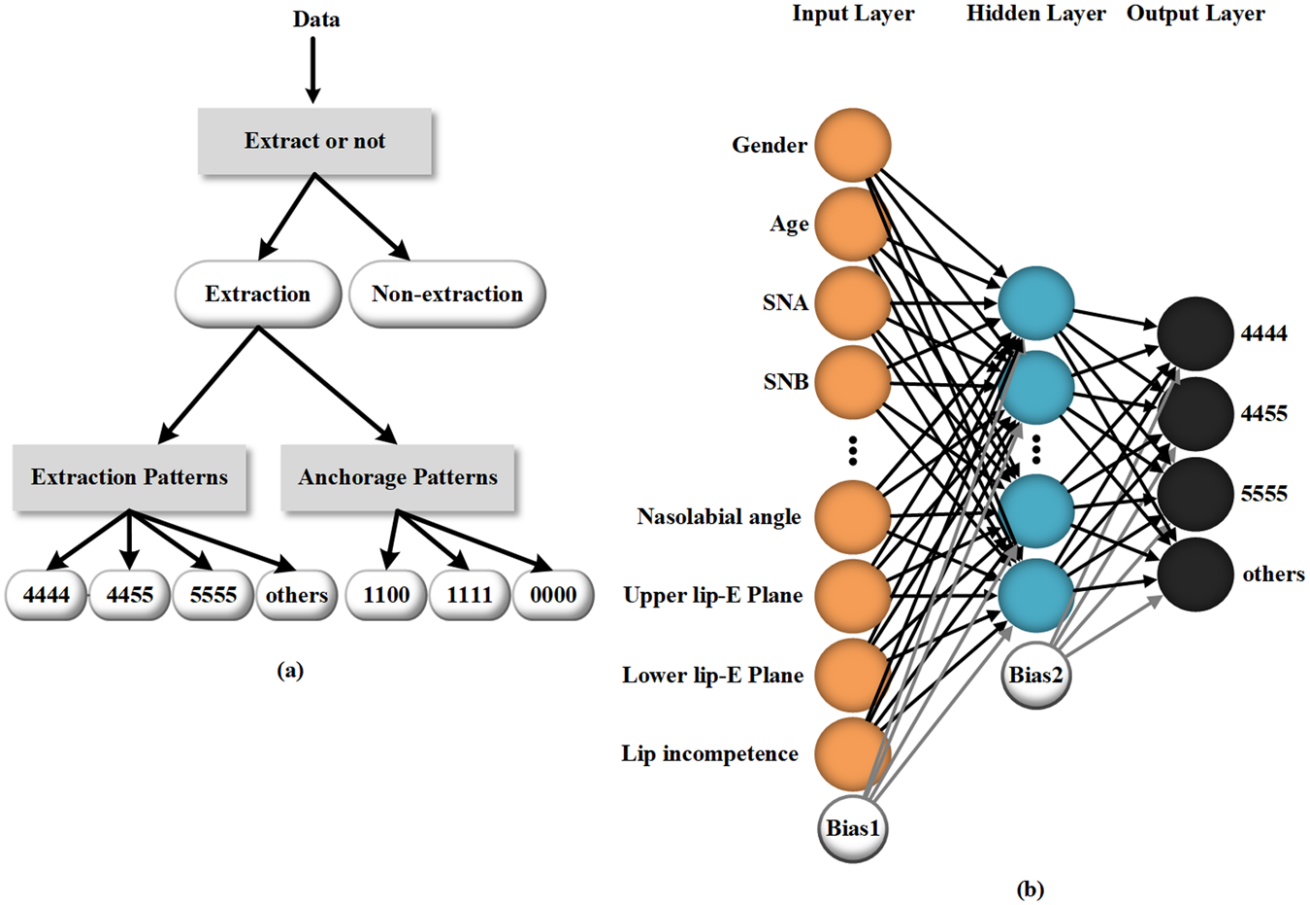
(**a**) The data processing flow chart; (**b**) structure of the neural network to predict the extraction patterns. The network structure is a three-layer fully connected multilayer perceptron consisting of 24 input nodes, 10 hidden nodes, and 4 output nodes.

Three neural networks are trained with 302 cases from the Department of Orthodontics, West China Hospital of Stomatology. As shown in Fig. 1a, the first neural network determines whether a patient needs tooth extraction. If the patient needs extraction, the second and third neural networks then predict the specific extraction pattern and anchorage pattern, respectively. The network to determine extraction patterns, for example, is a three-layer fully connected multilayer perceptron (MLP), as shown in Fig. 1b, which consists of 24 input nodes, 10 hidden nodes, and 4 output nodes. The other two neural networks share the same model structure as this network but have different numbers of output nodes. The extraction-nonextraction neural network has 2 output nodes, and the anchorage patterns neural network has 3. The trained neural network models are provided in the Supplementary Information, together with the demonstration of the treatment planning process.

### The accuracies of the ANNs

**Figure 2:**
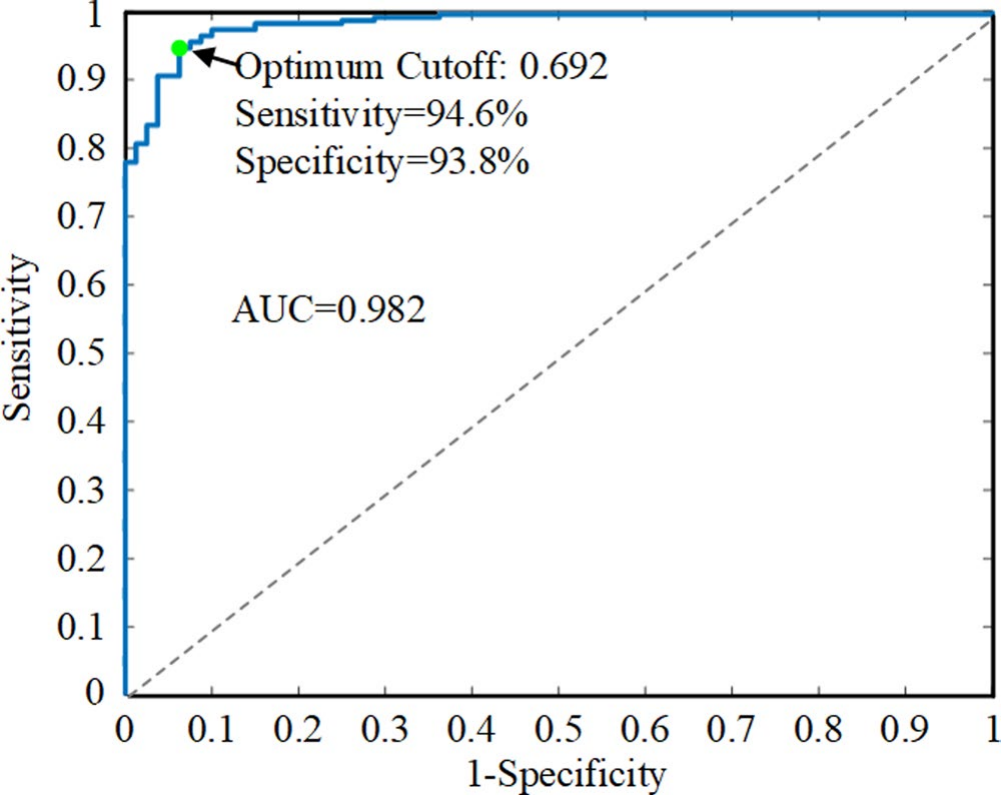
The ROC curve of the neural network to predict extraction. The model yields an AUC of 0.982 (95% CI 0.968–0.995). The optimum diagnostic cutoff value is 0.692, where the sensitivity of the model reaches 94.6% (95% CI 0.894–0.964) and the specificity reaches 93.8% (95% CI 0.870–0.984).

**Figure 3:**
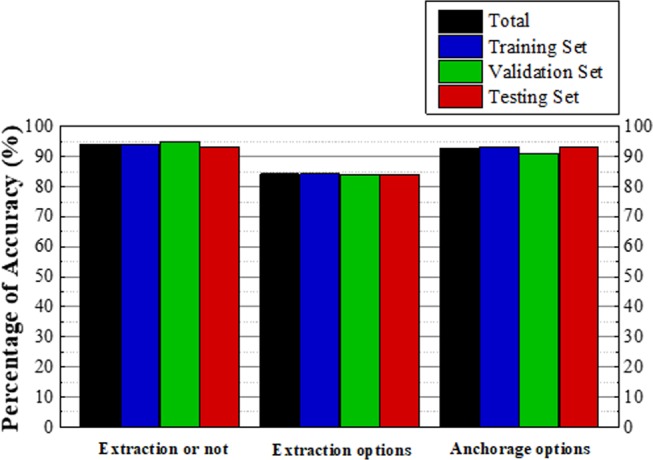
The accuracies of the ANNs. The accuracy of the extraction-nonextraction prediction is 94.0%, and the accuracies of the learning set, the validation set, and the test set are 94.0%, 95.0% and 93.3%, respectively. The predictive accuracy of the extraction patterns is 83.3%, and the accuracies of the learning set, validation set, and test set are 83.6%, 84.1%, and 81.8%, respectively. The overall accuracy of the anchorage patterns is 92.8%, and the accuracies of the learning set, validation set, and test set are 93.3%, 90.9%, and 93.2%, respectively.

As illustrated in Fig. 2, the receiver operating characteristic (ROC) curve shows the performance of the ANN on the extraction decision. The model yields an area under the curve (AUC) of 0.982 (95% CI 0.968–0.995). The closer the point on the ROC curve is to the upper left corner, the higher the accuracy of the model, and the point closest to the upper left corner is the best cutoff value with the least error. The optimum diagnostic cutoff value of this model is 0.692, with which the model reaches a sensitivity of 94.6% (95% CI 0.894–0.964) and a specificity of 93.8% (95% CI 0.870–0.984). If the prediction probability of a case for extraction is greater than 0.692, it will be diagnosed as an extraction case and passed to the other two models to determine the extraction pattern and anchorage pattern. Figure 3 shows the predictive accuracies of the ANNs. The accuracy of the extraction-nonextraction decision-making is 94.0%, and the accuracies of the learning set, validation set, and test set are 94.0%, 95.0%, and 93.3%, respectively. The predictive accuracy of the extraction patterns is 83.3%, and the accuracies of the learning set, validation set, and test set are 83.6%, 84.1%, and 81.8%, respectively. The overall accuracy of the anchorage patterns is 92.8%, and the accuracies of the learning set, validation set, and test set are 93.3%, 90.9%, and 93.2%, respectively. The decision-making of the extraction patterns is the most complicated part of the treatment planning, and different doctors may use different extraction patterns^16^. This explains why its prediction accuracy is lower than the other two parts of the treatment planning, and this gap is comparable to the results of another study^14^. Considering the subjectivity of decision-making on extraction patterns, the model offers several practicable alternatives for doctors to choose, which makes it more applicable.

### Relative contribution of features for planning decisions

In clinical practice, doctors may not always have access to all the required data used by ANNs. Therefore, investigating the contribution of all features for each decision part will be of practical importance. We used the PaD method^25^ to calculate the features’ relative contributions and ranked them in order. The results are illustrated in Table 1. With respect to extraction decision-making, features “crowding, upper arch” “crowding, lower arch” and “U1-NA°” are the three that contributed most. “ANB” “overbite” and “lip incompetence” are the three features that are most related to the prediction of extraction patterns. The three most important features for anchorage pattern determination by the model are the “curve of Spee”, “nasolabial angle” and “UL-EP”. The results demonstrate that these features are selected or “thought” as important by the models when making decisions. When the models make different predictions, they treat different features as the most important.

**Table 1:** Rank of the relative contribution of every feature.

Rank	Extraction or Non-extraction		Extraction Patterns		Anchorage Patterns	
	Features	Contribution	Features	Contribution	Features	Contribution
1	Crowding, Upper arch	85711.28105	ANB	118465.2103	Curve of Spee	18547.57224
2	Crowding, Lower arch	57736.71377	Overbite	114032.7377	Nasolabial angle	15026.78087
3	U1-NA°	15012.78143	Lip incompetence	41415.55706	UL-EP	14657.07879
4	UL-EP	4954.977657	U1-NA°	18085.09864	Overbite	12976.87069
5	LL-EP	4177.450009	Age	17473.38386	L1-NB°	7938.055508
6	L1-NB°	3401.656971	Curve of Spee	17302.13528	Profile	7680.987385
7	Lip incompetence	3286.95674	Crowding, Lower arch	16905.04732	FMIA	7414.201627
8	Overbite	2942.413326	Nasolabial angle	13745.41489	Age	6187.604495
9	Molar Relationship	1417.233	Overjet	13667.56045	LL-EP	2391.518896
10	Age	589.4921089	Molar Relationship	8798.718	ANB	2347.936804
11	S-Go/N-Me	588.7796549	U1-NA(mm)	7288.337735	S-Go/N-Me	1974.048274
12	Nasolabial angle	555.4425076	L1-NB(mm)	6406.556758	U1-NA°	1720.918869
13	Profile	343.9300483	UL-EP	5119.782824	Crowding, Lower arch	1467.612
14	Sex	307.2066295	FMIA	4089.027359	Lip incompetence	903.411461
15	U1-NA(mm)	208.265139	SNA	4056.661967	SNB	684.2875872
16	IMPA	150.1288817	Crowding, Upper arch	2949.948301	Sex	336.7110245
17	SNA	100.592332	FMA	1812.552422	U1-NA(mm)	258.1374941
18	FMIA	67.34319702	Profile	1437.646045	Molar Relationship	236.3783781
19	Overjet	57.1657521	SNB	1270.734428	IMPA	151.8654525
20	Curve of Spee	34.93226494	Sex	983.4392023	Crowding, Upper arch	106.1314566
21	SNB	5.236208966	IMPA	786.2672013	FMA	91.21057528
22	L1-NB(mm)	4.394215177	LL-EP	262.3915409	SNA	82.06470102
23	ANB	3.231616444	L1-NB°	93.51224553	L1-NB(mm)	34.28285845
24	FMA	1.03176361	S-Go/N-Me	4.960991795	Overjet	16.99804082

### Comparison of different complement methods

In the present study, we took a step further to investigate when the data of the most important features are missing, whether the impact of data loss on accuracies can be reduced or minimized by complement methods^21,23,24,26^. The complement effects are displayed in Table 2. The results show that the average value method has better performance than *k-*NN when dealing with discrete features regarding “lip incompetence” and “nasolabial angle”. The accuracies of the four traditional methods (average value, frequent value, specified value and median value methods) are consistent for processing the “nasolabial angle”. When dealing with continuous variables, *k-*NN performs better than the other methods, except for “crowding, lower arch”. With respect to the three features that the models “consider” to make the largest contribution to the treatment decisions, *k-*NN has the best complement performance. The effect of 2-*k-*NN is similar to that of 3-*k-*NN, in general.

**Table 2:** Complement effects of different methods on important features with missing data.

Complement methods	Average Value	Frequent Value	Specified Value	Median Value	2-*k-NN*	3-*k-NN*
**Network**	**Extraction or Non-extraction (accuracy of complete data: 0.9404)**					
Crowding, Upper arch	0.8642	0.8113	0.8113	0.8742	0.8775	**0.8841**
Crowding, Lower arch	**0.8974**	0.8742	0.8709	0.8642	0.8808	0.8808
U1-NA°	0.9106	0.9073	0.8974	0.9007	**0.9238**	0.9172
**Network**	**Extraction Patterns (accuracy of complete data: 0.8333)**					
ANB	0.8198	0.8063	0.8198	0.8153	**0.8268**	**0.8268**
Overbite	0.8198	0.8153	0.8153	0.8108	**0.8288**	0.8243
Lip incompetence	**0.8288**	0.8243	0.8243	0.6982	0.8063	0.8018
**Network**	**Anchorage Patterns (accuracy of complete data: 0.9279)**					
Curve of Spee	0.8649	0.8694	0.8694	0.8649	**0.8874**	0.8829
Nasolabial angle	**0.8784**	**0.8784**	**0.8784**	**0.8784**	0.8468	0.8559
UL-EP	0.8739	0.8333	0.8739	0.8739	0.8694	**0.8919**

The highest complement accuracy for each feature is shown in bold.

## Discussion

**Figure 4:**
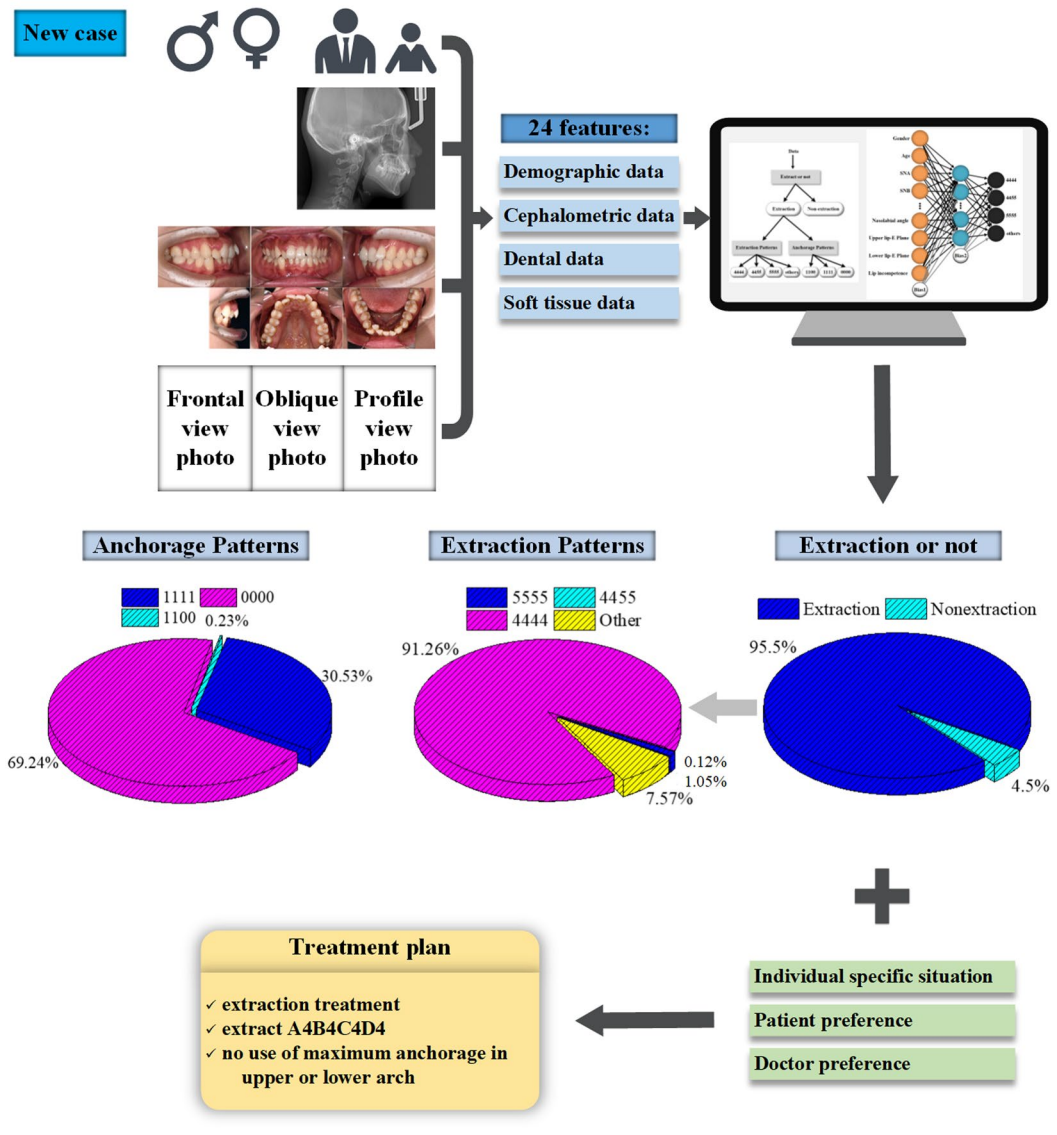
Clinical application illustration of the ANNs. The medical records of a new case were collected, and 24 input features, including demographic data, cephalometric data, dental data and soft tissue data, were extracted for neural network prediction. The extraction probability (0.955) was higher than 0.692; thus, it was determined as an extraction case and was passed to the other two networks. The other networks output the feasibilities of different extraction patterns and anchorage patterns. The doctor evaluated these treatment options, took other aspects into account, and finally determined an effective treatment plan.

In this study, we propose an MLP-based classifier to analyze patients’ medical records and output both a recommended treatment plan and the feasibilities of various treatment plans regarding the aspects of extraction *vs*. nonextraction, extraction patterns and anchorage patterns. The feature importance is calculated and ranked, and we compared the effects of different complement methods. Since there can be several feasible treatment plans regarding a certain case, the output probabilities of extraction and anchorage patterns offer users considerable flexibility as well as guidance. The users can review the recommended plan, compare various treatment options, take other aspects into account, and finally, develop a standardized, accurate, and effective treatment plan. Figure 4 illustrates the clinical application process of the ANNs. An example patient was used for demonstration. Informed consent to publish the information and images in an online open-access publication was obtained, and the example patient’s medical data (patient A) can be found in the Supplementary Note 2.

Our neural network models have better performance compared with previous prediction methods. The predictive accuracy of extraction reached 94.0%, higher than the other prediction models^13,27^, and made a one percent improvement on the 93% accuracy of Jung’s study^14^. The AUC of 0.9815 is also higher than the result of 0.904 in a previous study based on the CBES and the *k-*NN algorithm^9^. For the most complex part of extraction pattern prediction, the model reached 84.2% accuracy, similar to the result of Jung’s model^14^, which is also based on a neural network. In addition, this study is the first to make a prediction of the use of maximum anchorage by an ANN, to the best of our knowledge. The accuracy was 92.8%, suggesting the potential of ANN in assisting orthodontists in making more detailed treatment plans.

We used the PaD method to study the contribution of features to the outcome and ranked them in order. Xie^13^ used the “weights” method in a previous study and investigated the connection strengths of each neuron in the input layer with each neuron in the hidden layer to represent the contribution of every input index. They used different input indexes and different methods to calculate the contribution, and they found that “anterior teeth uncovered by incompetent lips” and “IMPA” were the two indexes that presented the largest contributions to the extraction decision vs. nonextraction. The “weights” method allows for a good classification of the input features but lacks stability^25^. The PaD method presents more complete results and makes a more severe discrimination between minor and major contributing variables in comparison to the “weights”^25,28^.

It is also the first time to investigate the effect of complement methods in similar studies. Our results suggested that the average value method outperformed *k-*NN when dealing with discrete variables, but *k-*NN performed better for the continuous variable. Meanwhile, *k-*NN is also a better choice when the data of the most important feature for each output decision is missing. This research contributes to the handling of missing data and can make the model more applicable.

It takes a relatively long time for orthodontists to accumulate experience. Doctors with less experience often require consultation with experts. Since medical developments are uneven and severely affected by economic conditions, expert consultation is especially deficient in areas with poor medical conditions. The proposed ANN system can not only assist less-experienced orthodontists and students in learning but also help patients obtain a clear understanding of their treatment plans.

## Methods

### Cases Collection

A total of 302 patients who received orthodontic treatment at the Department of Orthodontics, West China Hospital of Stomatology in Chengdu, China, from 2014 to 2018 were included in this study. The inclusion criteria were fixed labial appliance patients with full permanent dentition (except for second or third molars) without functional appliance treatment or orthognathic surgery. Their medical records before orthodontic treatment were collected, including demographic information, extraoral photos, intraoral photos, pretreatment dental casts and lateral cephalometric measurements^6,29^. Twenty-four commonly used feature variables were extracted from these clinical records as input features. The input features were preprocessed to ensure that all of them were quantified before being used for model training. Nonquantitative data were converted into numerical values by the encoding method. Supplementary Table S3 shows the detailed features used in the ANNs and how the nonquantitative data were encoded. All treatment planning was carefully performed by Dr. Zhao and Dr. Tang, who are both orthodontic specialists and have 26 and 12 years of clinical work experience, respectively. This study was approved by the West China Hospital of Stomatology Institutional Review Board (WCHSIRB-D-2018-094). Informed consent was obtained from all participants or their legal guardians. Informed consent for publication of the medical records of four example patients in an online open-access publication was also obtained. All experiments were performed in accordance with relevant guidelines and regulations.

### The composition of the cases and datasets

Among the total population, 222 persons were extraction cases, accounting for 73.5%, and the other 80 persons were nonextraction cases, accounting for 26.5%. The tooth extraction patterns were divided into four types: maxillary and mandibular first premolar extraction (4444), maxillary first premolar and mandibular second premolar extraction (4455), maxillary and mandibular second premolar extraction (5555) and other extraction patterns including only maxillary first premolar extraction, maxillary second premolar and mandibular first premolar extraction. These four patterns comprise 41.9%, 19.8%, 18.5% and 18.5% of the extraction cases, respectively. The anchorage patterns included three types, i.e., maxillary maximum anchorage (1100), maxillary and mandibular maximum anchorage (1111), and no use of maximum anchorage (0000), accounting for 29.7%, 21.6% and 48.6% of the extraction cases, respectively. Descriptions of the extraction patterns and anchorage patterns are shown in Table 3.

**Table 3:** Descriptions of the extraction patterns and anchorage patterns.

Pattern		Description
Extraction Patterns	4444	maxillary and mandibular first premolar extraction
	4455	maxillary first premolar and mandibular second premolar extraction
	5555	maxillary and mandibular second premolar extraction
	others	other extraction patterns including only maxillary first premolar extraction, maxillary second premolar and mandibular first premolar extraction
Anchorage Patterns	1100	maxillary maximum anchorage
	1111	maxillary and mandibular maximum anchorage
	0000	no use of maximum anchorage

The dataset is split into a training set, a validation set and a test set. The neural networks do not have access to the test set during the training process until the final evaluation of the accuracy. The reserve part of the dataset is split into a training set and a validation set with a ratio of 3/1, which is optimized according to the learning curve^30^. The training set is used to update the weights of the network. The validation set is used to avoid overfitting^13^. Considering that we had a smaller dataset, we used a greater percentage of data to test the models. Therefore, the training set, validation set and test set were set with a typical 60/20/20 split to maintain a balance between the sets. Cases with different tags were randomly distributed to the three datasets in each simulation so that the proportions of various cases are similar among the three sets, reducing the additional bias introduced by the data partitioning process. There are 222 extraction cases; thus, the 222 cases are used in the neural network models for predicting extraction patterns and anchorage patterns. The number and percentage of different kinds of treatment plans in each set are shown in Table 4.

**Table 4:** Number and percentage of different kinds of treatment plans in each set.

Sets	Extraction or Non-extraction			Extraction Patterns					Anchorage Patterns			
	Extraction (73.5%)	Nonextraction (26.5%)	Total (100%)	4444 (41.9%)	4455 (19.8%)	5555 (18.5%)	Others (18.5%)	Total (100%)	1100 (29.7%)	1111 (21.6%)	0000 (48.6%)	Total (100%)
Training (60%)	134	48	182	57	26	25	26	134	40	28	66	134
Validation (20%)	44	16	60	18	9	8	9	44	13	10	21	44
Test (20%)	44	16	60	18	9	8	9	44	13	10	21	44
Total (100%)	222	80	302	93	44	41	44	222	66	48	108	222

### Network models

All three neural networks used in this work are three-layer MLPs. Each MLP consists of three full connection layers. The MLP used to determine extraction patterns is illustrated in Fig. 1b. The activation function of the hidden layer is tanh. A *softmax* layer of 4 outputs is applied at the end of the model^31,32^. The cross-entropy^33,34^ *CE*_tanh_ is given by

1 $$CE=-\,t\ast \mathrm{log}(y)-(1-t)\ast \mathrm{log}(1-y)$$

where *t* is the target value and *y* is the output of the MLP. Equation (1) returns a numerical value approaching infinity, which heavily penalizes output when *y* approaches −1 or 1. *CE*_tanh_ approaches its minimum value when *y* approaches *t*. The weight and bias values are updated according to the scaled conjugate gradient method^35^. Although minimizing *CE*_tanh_ leads to a good accuracy of classification, considerably minimizing *CE*_tanh_ may cause overfitting. The dropout method is used to prevent overfitting^36,37^. The detailed training setting including learning rate, number of epochs, batch size, *et al*., are provided in Supplementary Note 4.

For the extraction prediction, the model outputs a probability of extraction. We define a determination of extraction treatment for each case as the probability of extraction being higher than a cutoff value. The algorithm computes sensitivity and specificity by testing a variety of cutoff values. Varying the cutoff point in the interval 0–1 generates a conventional ROC curve. Youden’s index^38^ is applied to obtain the optimum cutoff. If the probability is higher than the optimum cutoff, the case will be passed to the prediction of extraction patterns and anchorage patterns.

### Relative contribution calculation of features and complement of the missing data

The PaD method, which is supposed to be the most useful method in giving the relative contribution and the contribution profile of the input factors, was used to evaluate the relative contribution calculation of the input features. The PaD method computes the partial derivatives of the ANN’s output with respect to the input to obtain the profile of the variations of the output for small changes of one input variable. For a network with *n*_*i*_ inputs (where *i* represents the feature index and *i* = 1, 2, …, 24 in this work), one hidden tanh layer with *n*_*h*_ neurons, and no outputs, the partial derivatives of the output *y*_*j*_ with respect to input *x*_*j*_ (where *j* represents the case index and *j* = 1, 2, …, 302 in this work) are:

2 $${d}_{ij}={S}_{j}\sum _{h=1}^{{n}_{h}}{w}_{ho}(1-{I}_{hj}^{2}){w}_{ih}$$

where *S*_*j*_ is the derivative of the output neuron with respect to the input, which is the weights between the output neuron and *h*_*th*_ hidden neuron, *I*_*hj*_ is the output of the *h*_*th*_ hidden neuron, and *w*_*ih*_ is the weights between the *i*_*th*_ input neuron and the *h*_*th*_ hidden neuron.

Then, the relative contribution of the ANN’s output to the dataset with respect to the *i*_*th*_ input feature can be calculated by a sum of the square partial derivatives as:

3 $$SS{D}_{i}=\sum _{j=1}^{N}{d}_{ij}^{2}$$

where *N* is the data size and equals 302 in this work. The SSD values enable direct access to the influence of each input variable on the output.

We use the average value method, frequent value method, specific value method, median value method, and *k-*NN method to study their complement effects. The four traditional methods complement the missing data with the average value, the frequent value, the specified value (standard value of normal population), and the median value. The *k-*NN method is a method to look for the new case’s nearest neighbors from the complete cases and use an estimated value to replace the missing data^26,39^. This value is the weighted average of the values of its *k* nearest neighbors. We used 2-*k-*NN (2 nearest neighbors) and 3-*k-*NN (3 nearest neighbors) in this study. Each neighbor is given a weight of *1/d*, where *d* is the distance to the neighbor. The neighbors are taken from the dataset for which the object property value is known.

## Supplementary information

Supplementary Information

## Data Availability

The present neural network models, medical data of example patients, features descriptions and training setting are provided in the Supplementary Information.
