# Supplementary material for: Orthodontic Treatment Planning based on Artificial Neural Networks
Source: Sci Rep. 2019 Feb 14;9:2037. doi: 10.1038/s41598-018-38439-w (PMC6375961; doi:10.1038/s41598-018-38439-w)
Supplement: Supplementary file 1 — Supplementary Information [file 41598_2018_38439_MOESM1_ESM.zip › Supplementary material.docx]

**Orthodontic Treatment Planning based on Artificial Neural Networks**

**Peilin Li^1^, Deyu Kong^2^, Tian Tang^1^, Di Su^1^, Pu Yang^1^, Huixia Wang^1^, Zhihe Zhao^1,*^, and Yang Liu^2,*^**

^1^State Key Laboratory of Oral Diseases & National Clinical Research Center for Oral Diseases & Department of Orthodontics, West China Hospital of Stomatology, Sichuan University, Chengdu, 610041, P.R. China.

^2^State Key Laboratory of Electronic Thin Films and Integrated Devices, University of Electronic Science and Technology of China, Chengdu, 610054, P. R. China.

**Corresponding authors:**

Zhihe Zhao: [zhzhao@scu.edu.cn](mailto:zhzhao@scu.edu.cn); Yang Liu: [yliu1975@uestc.edu.cn](mailto:yliu1975@uestc.edu.cn)

# SUPPLEMENTARY INFORMATION

**Supplementary Note 1. Treatment planning with the neural networks**

The trained networks are uploaded as supplementary information named as “**Networks.mat**”. The networks are trained and used with Matlab^®^ R2017a. To make a treatment plan with the present networks, “**Networks.mat**” should be loaded first. An example patient named “patient” will also be loaded when loading the networks. The data of the new patient should be strictly organized in order as demonstrated in **Table 1**, which is a column vector of 24 elements.

**Supplementary Table S1** presents some help for operation. The corresponding commands should be entered into the command window of Matlab for the certain purposes.

| Commands | Description |
| --- | --- |
| load (‘Networks.mat’) | To load the “Networks.mat” |
| sim(net_ext, patient) | To diagnosis extraction-nonextraction for “patient” |
| sim(net_ext_pattern, patient) | To diagnosis extraction pattern for “patient” |
| sim(net_anc_pattern, patient) | To diagnosis anchorage pattern for “patient” |

**Supplementary Table S1.** Commands for use.

**Supplementary Note 2. Medical data of example patients**

| **Features** | **Patient A** | **Patient B** | **Patient C** | **Patient D** |
| --- | --- | --- | --- | --- |
| Gender | 0 | 0 | 0 | 0 |
| Age | 24 | 26 | 24 | 14 |
| SNA | 82.3 | 79.9 | 79.1 | 80.6 |
| SNB | 76.1 | 74.2 | 77.7 | 79.7 |
| ANB | 6.2 | 5.7 | 1.2 | 0.9 |
| S-Go/N-Me | 61.4 | 58.8 | 60.6 | 69.1 |
| U1-NA | 2.6 | 4.2 | 5.5 | 9.8 |
| U1-NA | 21.4 | 31.3 | 33.2 | 45.9 |
| L1-NB | 6.9 | 8.8 | 5.5 | 4.4 |
| L1-NB | 28.1 | 30.3 | 23.6 | 19.8 |
| FMA | 30.9 | 36.2 | 31.8 | 16.9 |
| IMPA | 89.8 | 92.2 | 83.0 | 91.5 |
| FMIA | 59.3 | 51.6 | 55.3 | 71.5 |
| Overbite | 4.9 | 0.0 | 0.6 | 1.9 |
| Overjet | 4.7 | 1.5 | 1.0 | 6.8 |
| Crowding, Upper arch | 4.5 | 11.0 | -1.0 | 2.5 |
| Crowding, Lower arch | 8.0 | 7.0 | 2.0 | 5.0 |
| Curve of Spee | 2.0 | 1.5 | 3.0 | 2.0 |
| Molar Relationship, Left/Right | (0, 0) | (1, 1) | (0, -1) | (0, 0) |
| Profile | 1 | 1 | 0 | 1 |
| Nasolabial angle | 0 | 0 | 0 | 0 |
| Upper lip-E Plane | -1.3 | 0.3 | -2.0 | -2.0 |
| Lower lip-E Plane | 1.4 | 3.5 | -1.0 | -0.3 |
| Lip incompetence | 0 | 0 | 0 | 0 |
| **Extraction probabilities** | 0.955 | 1.000 | 0.161 | 0.994 |
| **Treatment plans** | | | | |
| Extraction-nonextraction | 1 | 1 | 0 | 1 |
| Extraction patterns | 4444 | 4444 | - | 5555 |
| Anchorage patterns | 0000 | 1100 | - | 0000 |

**Supplementary Table S2.** The medical data of example patients.

**Supplementary Note 3. The detailed features used in the ANNs.**

| **Features** | **Input Type** | **Units** | **Details** | |  |
| --- | --- | --- | --- | --- | --- |
| Gender | Discrete | - | Females are 0 and males are 1. | |  |
| Age | Integral | year | The age range is 9-40, with a mean age of 17.16±5.71 | |  |
|  |  |  |  |  |  |
| SNA | Continuous | ° | Reflcting relationship of the maxilla to the cranial base. | |  |
| SNB | Continuous | ° | Reflcting relationship of the mandible to the cranial base. | |  |
| ANB | Continuous | ° | Indicating the magnitude of the skeletal jaw discrepancy. | |  |
| S-Go/N-Me | Continuous | % | Ratio of posterior facial height to anterior facial height, indicating growth pattern. | |  |
| U1-NA | Continuous | mm | Establishing the position of the maxillary dentition relative to the maxilla, including the distance of the labial surface of upper incisor in front of NA line and the inclination of it to the line. | |  |
| U1-NA | Continuous | ° |  |  |  |
| L1-NB | Continuous | mm | Establishing the position of the mandibular dentition relative to the mandible, including the distance of the labial surface of lower incisor in front of NB line and the inclination of it to the line. | |  |
| L1-NB | Continuous | ° |  |  |  |
| FMA | Continuous | ° | Mandibular plane angle, i.e. the angle between the Frankfort plane and the mandibular plane. | |  |
| IMPA | Continuous | ° | The intersection of the long axis of the lower central incisor and the mandibular plane. | |  |
| FMIA | Continuous | ° | The intersection of the long axis of the lower central incisor and the Feankfort plane. | |  |
| Overbite | Continuous | mm | The vertical distance between the edges of upper and lower incisors. | |  |
| Overjet | Continuous | mm | The projection distance of the upper and lower central incisors' cutting edges on the occlusal plane. | |  |
| Crowding, Upper arch | Continuous | mm | The discrepancy between space required and space available in the upper arch. | |  |
| Crowding, Lower arch | Continuous | mm | The discrepancy between space required and space available in the lower arch. | |  |
| Curve of Spee | Continuous | mm | The anatomic curvature of the mandibular occlusal plane. | |  |
| Molar Relationship, Left/Right | Discrete | - | Class I, II, III molar relationships are labled as 0, 1 and -1, respectively. The first number indicates molar relationship on the left side, the latter indicates the right side. E.g. (0, 1) means that molar relationships are Class I on the left side and Class II on the right side. | |  |
| Profile | Tripartite | - | Straight profiles are 0, convex profiles are 1 and concave profile are -1. | |  |
|  |  |  |  |  |  |
| Nasolabial angle | Tripartite | - | Normal nasolabial angles are 0, smaller angles are -1 and larger angles are 1. | |  |
|  |  |  |  |  |  |
| Upper lip-E Plane | Continuous | mm | The distance of upper lip to aesthetic plane. | |  |
| Lower lip-E Plane | Continuous | mm | The distance of lower lip to aesthetic plane. | |  |
| Lip incompetence | Binary | - | Lip competence is 0 and lip imcompetence is 1. | |  |
|  |  |  |  | |  |
| **Supplementary Table S3.** The detailed features used in the ANNs. | | | |  |  |
|  |  |  |  | |  |

**Supplementary Note 4. Training Setting**

The MLP is trained with Matlab (Version R2017a) Neural Pattern Recognition App. The Matlab training script is uploaded as Supplementary Material with file name “TrainScript.m”.

**Learning Rate**

The initial learning rate is set as 0.01.

**Number of Epoch**

The total epoch number is 33. The best epoch is Epoch 27. The training state is shown in Supplementary Figure 1.

**Supplementary Figure S1.** The MLP’s training state (gradient and the number of consecutive). The training process stops if validation performance has increased more than 6 times since the last time it decreased.

**Training Function**

We use scaled conjugate gradient back propagation (trainscg) training function to update weight and bias values according to the scaled conjugate gradient method.

Training stops when any of these conditions occurs:

1. The maximum number of epochs (repetitions) is reached.

2. The maximum amount of time is exceeded.

3. Performance is minimized to the goal.

4. The performance gradient falls below min_grad.

5. Validation performance has increased more than max_fail times since the last time it decreased.

As shown in Supplementary Figure S1, our training early stopped as Condition 5 occurs.

| **Parameter** | **Value** | **Description** |
| --- | --- | --- |
| net.trainParam.epochs | 1000 | Maximum number of epochs to train |
| net.trainParam.showWindow | true | Show training GUI |
| net.trainParam.goal | 0 | Performance goal |
| net.trainParam.time | inf | Maximum time to train in seconds |
| net.trainParam.min_grad | 1e-6 | Minimum performance gradient |
| net.trainParam.max_fail | 6 | Maximum validation failures |

**Supplementary Table S4. Option Parameters of Trainscg.**

**Batch Size**

Full batch learning method is used. The batch size equals to the training set size.

**Initial Method**

The Nguyen-Widrow method is used, which generates initial weight and bias values for a layer so that the active regions of the layer’s neurons are distributed approximately evenly over the input space.
